# In vitro and in ovo impact of the ionic dissolution products of boron-doped bioactive silicate glasses on cell viability, osteogenesis and angiogenesis

**DOI:** 10.1038/s41598-022-12430-y

**Published:** 2022-05-20

**Authors:** Simon Decker, Marcela Arango-Ospina, Felix Rehder, Arash Moghaddam, Rolf Simon, Christian Merle, Tobias Renkawitz, Aldo R. Boccaccini, Fabian Westhauser

**Affiliations:** 1 Department of Orthopaedics, Heidelberg University Hospital, Schlierbacher Landstraße 200a, 69118 Heidelberg, Germany; 2 Institute of Biomaterials, University of Erlangen-Nuremberg, Cauerstr. 6, 91058 Erlangen, Germany; 3 Orthopedic and Trauma Surgery, Frohsinnstraße 12, 63739 Aschaffenburg, Germany

**Keywords:** Biomedical materials, Glasses, Materials science

## Abstract

Due to the pivotal role of angiogenesis in bone regeneration, the angiogenic properties of biomaterials are of high importance since they directly correlate with the biomaterials’ osteogenic potential via ‘angiogenic-osteogenic coupling’ mechanisms. The impact of bioactive glasses (BGs) on vascularization can be tailored by incorporation of biologically active ions such as boron (B). Based on the ICIE16-BG composition (in mol%: 49.5 SiO_2_, 36.3 CaO, 6.6 Na_2_O, 1.1 P_2_O_5_, 6.6 K_2_O), three B-doped BGs have been developed (compositions in mol%: 46.5/45.5/41.5 SiO_2_, 36.3 CaO, 6.6 Na_2_O, 1.1 P_2_O_5_, 6.6 K_2_O, 3/4/8 B_2_O_3_). The influence of B-doping on the viability, cellular osteogenic differentiation and expression of osteogenic and angiogenic marker genes of bone marrow-derived mesenchymal stromal cells (BMSCs) was analyzed by cultivating BMSCs in presence of the BGs’ ionic dissolution products (IDPs). Furthermore, the influence of the IDPs on angiogenesis was evaluated in ovo using a chorioallantoic membrane (CAM) assay. The influence of B-doped BGs on BMSC viability was dose-dependent, with higher B concentrations showing limited negative effects. B-doping led to a slight stimulation of osteogenesis and angiogenesis in vitro. In contrast to that, B-doping significantly enhanced vascularization in ovo, especially in higher concentrations. Differences between the results of the in vitro and in ovo part of this study might be explained via the different importance of vascularization in both settings. The implementation of new experimental models that cover the ‘angiogenic-osteogenic coupling’ mechanisms is highly relevant, for instance via extending the application of the CAM assay from solely angiogenic to angiogenic and osteogenic purposes.

## Introduction

Since the development of the 45S5-bioactive glass (BG) composition by Hench and colleagues^1^, BGs have gained relevance as bone substituting materials and, more recently, in bone tissue engineering (BTE)^2^. Over the years, BG compositions were constantly altered to optimize their mechanical and biological properties, eventually leading to development of new BGs^2,3^. As such, the ICIE16-BG (in mol%: 49.5 SiO_2_, 36.3 CaO, 6.6 Na_2_O, 1.1 P_2_O_5_, 6.6 K_2_O) was introduced by Elgayar and co-workers in 2004^4^, showing good biocompatibility and enhanced osteogenic properties when compared to 45S5-BG in vitro^5^. From a materials perspective, ICIE16-BG exhibits favorable processing properties to fabricate 3D scaffolds, while maintaining a good bioactivity^6,7^. Moreover, ICIE16-BG allows tailoring its (biological) characteristics through incorporation of ions with therapeutic effects^7,8^.

Boron (B) is a promising candidate ion since a positive influence on healthy bone development and regeneration has been reported^9–11^. Furthermore, B is known for its positive effects on wound healing and angiogenesis^12–15^. As angiogenesis plays a pivotal role in both osteogenesis and bone regeneration in a 3D tissue environment^16–18^, pro-angiogenic properties are considered especially attractive and relevant for BG dopant ions^19^. However, most in vitro studies suggest that B is cytotoxic in high concentrations, therefore beneficial biological effects of B seem to be limited to a specific therapeutic concentration window^20–22^.

While doping different BGs with B led to increased conversion of BGs to hydroxyapatite^21^, improved osteogenic differentiation^23,24^ and enhanced bone formation in vivo^25^, there is some evidence concerning the potentially pro-angiogenic properties of B-doped BGs, especially in vivo, available^26^. In an in vitro study with human umbilical vein endothelial cells (HUVECs), doping BGs with B increased tubulogenesis and secretion of pro-angiogenic factors^27^, in studies with ST-2 cells the release of the pro-angiogenic vascular endothelial growth factor (VEGF) in presence of B-containing BGs was enhanced^28,29^. In mice, B-doped BGs increased angiogenic gene expression patterns and osteoid formation^22^, whereas angiogenesis was stimulated by ionic dissolution products (IDPs) of a 45S5-BG doped with B in a chorioallantoic membrane (CAM) assay in ovo^17^.

So far, the osteogenic and angiogenic properties as well as the biocompatibility of B-doped ICIE16-BGs have not yet been investigated. Therefore, the impact of IDPs of 3B-BG, 4B-BG and 8B-BG (compositions in mol%: 46.5/45.5/41.5 SiO_2_, 36.3 CaO, 6.6 Na_2_O, 1.1 P_2_O_5_, 6.6 K_2_O, 3/4/8 B_2_O_3_) on the viability, cellular osteogenic differentiation and expression of osteogenic and angiogenic marker genes of bone marrow-derived mesenchymal stromal cells (BMSCs) was evaluated and compared to IDPs of the undoped ICIE16-BG in this study. Subsequently, the influence of the IDPs of the respective BGs on angiogenesis was evaluated in ovo using a CAM assay as an alternative method to common in vivo animal experiments^30,31^.

## Materials and methods

### BG production and characterization

The BGs based on the ICIE16-BG composition were produced via the melt-quench route from analytical grade reagents including, NaCO_3_ (Honeywell Fluka, Steinheim, Germany), K_2_CO_3_ (Alfa Aesar, Erlenbachweg, Germany), CaCO_3_ (Honeywell Fluka), CaHPO_4_·2H_2_O (Acros Organics, Geel, Belgium), H_3_BO_3_ (Merck, Darmstadt, Germany) and commercial-grade Belgian quartz sand (SiO_2_). The glasses were melted in Pt crucibles for 1.5 h at a melting temperature of 1420 °C for the reference ICIE16-BG and 1200 °C for the B-doped BGs. The casting was performed in graphite molds followed by an annealing process at 520 °C for 1 h. Moreover, a second melting was carried out to ensure homogeneity. A Jaw Crusher (Retsch, Germany) was used to crush the BGs for the subsequent grinding process to obtain fine powders using a planetary ball mill (Retsch, Germany). A thermal treatment was carried out to sinter the BGs at a heating rate of 2 °C/min. The ICIE16-BG was sintered at 690 °C for 1.5 h and the B-doped BGs at 680 °C for 1.5 h (to simulate the heat-treatment schedule required to produce scaffolds by powder sintering). The sintered BGs were finally crushed, grounded to fine powder, and sieved using a 100-micron mesh sieve. The composition of the resulting BGs is given in Table 1.

**Table 1:** Chemical composition of BGs used in the experiments (in mol%).

	SiO_2_	CaO	P_2_O_5_	Na_2_O	K_2_O	B_2_O_3_
ICIE16-BG	49.46	36.27	1.07	6.6	6.6	–
3B-BG	46.46	36.27	1.07	6.6	6.6	3
4B-BG	45.46	36.27	1.07	6.6	6.6	4
8B-BG	41.46	36.27	1.07	6.6	6.6	8

Scanning electron microscopy (SEM, Auriga, Carl-Zeiss, Jena, Germany) was used to observe the morphology of the BG particles at a voltage of 1.5 kV, additionally the particle size of the granules was estimated from SEM pictures by measuring at least 100 particles using the software ImageJ (U.S. National Institutes of Health, Bethesda, MD, USA). To obtain the concentration of leaching ions from the BGs, samples were immersed in simulated body fluid (SBF), prepared as reported by Kokubo et al.^32^, in a concentration of 1.5 mg/ml and placed in an orbital shaker at 37 °C and 90 rpm agitation for 21 days, measurements were performed after 8 h, 1, 3, 7, 14 and 21 days. The supernatant of the samples was analyzed with an inductively coupled plasma-optical emission spectrometer (ICP-OES, PerkinElmer Optima 5300 DV, Shelton, CT, USA). Since the aim of the current work is the investigation of the impact of B-doped BGs’ IDPs on osteogenesis and angiogenesis, further characterization of the synthesized BGs, such as thermal properties or bioactivity is not discussed here, as this has been presented in detail in our previous work^8^.

### Study ethics and cell origin

BMSCs of a 20-year-old male patient undergoing surgery at the proximal femur at the Heidelberg Orthopedic University Hospital were harvested. The protocol of the study strictly followed the contents of the declaration of Helsinki in its present form. The patient’s written informed consent was obtained prior to cell collection. The responsible ethics committee of the Medical Faculty of Heidelberg University approved the use of the cells for the means of this study (S-340/2018).

### BMSC isolation and cultivation

The isolation of BMSCs was performed following a density gradient centrifugation protocol as published previously^33–35^. After extracting mononuclear cells from donor bone marrow, cell cultivation was performed in 0.1% gelatin (Sigma Aldrich, Steinheim, Germany) coated T75 cell culture flasks (Sarstedt, Nümbrecht, Germany) in expansion medium (EM), consisting of Dulbecco’s modified Eagle’s medium (DMEM) high glucose, 12.5% fetal calf serum (FCS), 2 mM L-glutamine, 1% non-essential amino acids (NEAA), 50 µM β-mercaptoethanol (all Life Technologies, Darmstadt, Germany), 100 µg/ml penicillin/streptomycin (Sigma-Aldrich) and 4 ng/ml fibroblast growth factor 2 (Abcam, Cambridge, U.K.) under standard cell culture conditions (37 °C and 5% CO_2_ in a humidified atmosphere). Medium was exchanged after 24 h to discard non-adherent cells and subsequently twice per week. At 80% confluency, cells were passaged and stored in liquid nitrogen. The experiments were conducted with BMSCs in passage 3.

### General experimental design: overview

The study was divided in an in vitro and an in ovo part. In the in vitro part, the influence of IDPs of the BGs on BMSCs was assessed using an indirect cultivation setting as published previously^36,37^. The different BGs were added to cell culture medium (CCM; DMEM high glucose, 10% FCS, 100 µg/ml penicillin/streptomycin) at a concentration of 1 mg/ml and incubated under standard cell culture conditions. After three days, medium conditioned with the BGs’ IDPs was collected. The volume of collected CCM was replaced by the same amount of fresh CCM. BMSCs were seeded in filtered IDPs-containing CCM at a density of 18,400 cells/cm^2^ in 24- or 96-well-plates (both Sarstedt), depending on the type of assay. A control group was seeded in regular, IDPs-free CCM. Medium exchange was performed twice a week. After 1 (D1), 3, (D3), 7 (D7), 10 (D10), 14 (D14) and 21 (D21) days, the various assessment methods evaluating the influence of the BGs’ IDPs on cell viability, osteogenic differentiation and angiogenesis were conducted.

In the in ovo part of this study, a CAM assay was used to further assess the influence of the IDPs of the BGs on angiogenesis. The CAM assay is a well-known method in angiogenesis research and represents an attractive alternative to in vivo animal experiments^30,31^. The indirect culture setting described above was used in this part of the study as well, IDP-conditioned medium was collected after seven days. Cells were transplanted in their respective IDPs-free or IDPs-containing medium onto the CAM and the angiogenic response was qualitatively evaluated after 1 (D1), 4 (D4) and 7 (D7) days. Transplants were resected 7 days post transplantation and quantitative analysis of the angiogenic response, as well as histologic evaluation followed.

### In vitro evaluation

#### Combined cell viability and alkaline phosphatase (ALP) activity assay

Cell viability and alkaline phosphatase (ALP) activity, a well-known marker of cellular osteogenic differentiation, were determined using a combined fluorescence-based assay following established protocols^38^. Due to its correlation with cell number and viability^5,39^, measurement of fluorescein diacetate (FDA) metabolization was used to assess cell viability. The conversion of the ALP-substrate 4-methylumbelliferyl phosphate (4-MUP) was measured, since it correlates directly with ALP activity^38^. In short, after discarding CCM and washing cells with Dulbecco’s phosphate-buffered saline (DPBS, Life Technologies), cells were stained with FDA substrate solution (0.1 mg/ml FDA (Sigma-Aldrich) in acetone (Carl Roth, Karlsruhe, Germany) 1:50 diluted in DPBS) at 37 °C for 5 min. Another washing step with DPBS followed before cells were lyzed with 0.5% Triton-X-100 (Sigma-Aldrich) at 37 °C for 5 min. Aliquots of the cell lysates were transferred to a white 96-well-plate (Kisker Biotech, Steinfurt, Germany), then 4-MUP substrate solution (100 µM 4-MUP (Life Technologies) in ALP assay buffer consisting of 75 mM TRIS pH 9.3, 1.5 mM MgCl_2_ and 0.15 mM ZnCl_2_ (all Carl Roth)) was added and incubated at 37 °C for 15 min. A fluorescence microplate reader (Wallac 1420 Victor 2; Perkin Elmer, Waltham, MA, USA) was used to determine the emerging fluorescence at 485/530 nm (ex/em) for FDA and at 360/440 nm (ex/em) for 4-MUP. ALP activity was normalized to FDA fluorescence intensity.

#### Qualitative analysis of cell morphology and viability

Visualization of cell morphology and viability was conducted using a fluorescence microscopy-based live/dead-assay. Propidium iodide (PI), which cannot pass viable cell membranes and therefore intercalates into DNA of compromised cells^5,40^ was applied to detect potentially remaining dead cells, whereas viable cells were visualized with FDA. After discarding CCM, staining solution composed of 8 µg/ml FDA and 20 µg/ml PI (Life Technologies) in DPBS was added and incubated at 37 °C for 5 min. Staining solution was disposed and cells were kept in DPBS for imaging with an Olympus IX-81 inverted fluorescence microscope (Olympus, Hamburg, Germany). Green (FDA) and red (PI) pictures were merged with ImageJ software.

#### Analysis of osteogenic and angiogenic marker gene expression via qPCR

To assess osteogenic differentiation on a genetic level, gene expression of relevant genes correlating with osteogenic differentiation, namely osteopontin (OPN), osteocalcin (OCN), and bone morphogenic protein-2 (BMP-2) was analyzed via qPCR. To assess the influence of the BGs’ IDPs on angiogenesis on a genetic level, the expression of the angiogenic marker genes angiopoietin-1 (ANGPT1), vascular endothelial growth factor A (VEGF-A) and endothelin 1 (EDN1) was analyzed as well. RNA-Isolation was conducted using PureLink RNA Mini Kit (Life Technologies) following the manufacturer’s protocol, then 100 ng of RNA were reversely transcribed into cDNA with High-Capacity RNA-to-cDNA-Kit (Life Technologies) according to the manufacturer’s instructions. qPCR was conducted in a LineGene 9600 Fluorescent Quantitative Detection System (Hangzhou Bioer Technology, Hangzhou, China) using PowerUp SYBR Green Master Mix (Life Technologies) and the primer pairs shown in Table 2. Gene expression was calculated using the ΔΔCt method: Analyzed genes were referred to tyrosine 3-monooxygenase/tryptophan 5-monooxygenase activation protein zeta (YWHAZ), which served as endogenous reference gene, followed by normalization to the control group. Measurements were performed in technical duplicates.

**Table 2:** Primer pairs used for qPCR. Tyrosine 3-monooxygenase/tryptophan 5-monooxygenase activation protein zeta (YWHAZ; reference gene), secreted phosphoprotein 1/osteopontin (SPP1/OPN), osteocalcin (OCN), bone morphogenic protein-2 (BMP-2), angiopoietin-1 (ANGPT1), vascular endothelial growth factor A (VEGF-A), endothelin 1 (EDN1).

Gene	Forward (5’ $\to$3’)	Reverse (5’ $\to$3’)
YWHAZ	TGC TTG CAT CCC ACA GAC TA	AGG CAG ACA ATG ACA GAC CA
OPN	GCT AAA CCC TGA CCC ATC TC	ATA ACT GTC CTT CCC ACG GC
OCN	ACC GAG ACA CCA TGA GAG CC	GCT TGG ACA CAA AGG CTG CAC
BMP-2	CAG ACC ACC GGT TGG AGA	CCA CTC GTT TCT GGT AGT TCT TC
ANGPT1	CCT GAT CTT ACA CGG TGC TGA TT	GTC CCG CAG TAT AGA ACA TTC CA
VEGF-A	GGG CAG AAT CAT CAC GAA G	ATC TGC ATG GTG ATG TTG GA
EDN1	AAG ACA AAC CAG GTC GGA GA	TGG AGG CTA TGG CTT CAG AC

### In ovo evaluation

#### The CAM assay

A CAM assay was used to assess the influence of the IDPs of the BGs on angiogenesis, based on a previously published protocol by Kunz et al., that has been adapted for the purposes of this study^41^. Fertilized white Leghorn chicken eggs were purchased from a local ecological hatchery (Geflügelzucht Hockenberger, Eppingen, Germany), the delivery day was defined as embryonic development day 0 (EDD 0). After cleaning with sterile water, the eggs were incubated in an upright position under permanent agitation in a humidified atmosphere at 37.8 °C. To prepare the eggs for transplantation, 3 ml albumin was removed under diaphanoscopy with a 20 gauge needle (Becton Dickinson, Heidelberg, Germany) at the wider end of the egg at EDD 4. Then, the CAM was exposed by cutting a window of approximately 1.5 cm diameter into the upper side of the egg. The cut-out eggshell was not removed, but used to cover the window and sealed with medical tape (Leukosilk; BSN medical, Hamburg, Germany) for further incubation. On EDD 9, a sterile silicone ring (Greiner Bio-One, Frickenhausen, Germany) was placed on the CAM and the area within the ring was gently lacerated with a 30 gauge needle (B. Braun, Melsungen, Germany) to prepare cell transplantation. Per egg, 1 × 10^6^ BMSCs were resuspended in 10 µl IDPs-conditioned or IDPs-free CCM, before being mixed with an equal volume of Cultrex BME Type 3 (Bio-Techne, Wiesbaden, Germany) matrix hydrogel. Cell suspensions were directly applied onto the CAM after mixing and transplantation day was set as day 0 (D0) of the experiment. To monitor the transplants on the CAM and their influence on the vessels, the CAMs were photographed after 1 (D1), 4 (D4) and 7 (D7) days. Seven days post transplantation (EDD 16, D7), chick embryos were euthanized by intravascular injection of 50 µl Narcoren® (Boehringer Ingelheim Vetmedica, Ingelheim, Germany) and transplants were resected. To quantitatively analyze the angiogenic response, resectates were imaged with a Zeiss Axioplan 2 microscope (Carl Zeiss, Oberkochen, Germany) directly after explantation. According to regional and federal regulations, ethical approval for animal experimentation is not required for utilization of the CAM assay.

#### Quantitative analysis of the angiogenic response

Quantitative analysis of the CAM’s angiogenic response to the transplants was performed using a modified version of the ‘vascular index’, as described by Barnhill et al.^42^. The area of the transplant, as well as a surrounding 1 mm annulus, defining the area of interest (AOI), were marked with GIMP (GNU Image Manipulation Program, Version 2.10.22). The number of vessels within the AOI was counted manually using Aperio ImageScope (Version 12.4.3.5008, Leica Biosystems, Nussloch, Germany) and normalized to the AOI, as measured with ImageJ.

#### Histologic processing and in situ hybridization

After CAM resectates were fixated and embedded in paraffin following established protocols^43^, in situ hybridization of repetitive species-specific genomic sequences was conducted to detect human BMSCs on the resectates, as described earlier^44^. In short, human lysozyme (hALU)-labeling was used to assess whether the transplanted human BMSCs are still present on the CAMs following the seven-day incubation period. Imaging was performed using a Zeiss Axioplan 2 microscope.

### Statistics

Statistical analyses were conducted with IBM SPSS Statistics (Version 25; IBM, Armonk, NY, USA). Values were compared using Kruskal–Wallis and Mann–Whitney-U test with *p* < 0.05 as the level of significance. Due to the study’s exploratory design, no correction for multiple testing was performed^45^. Graphs were designed with GraphPad Prism (Version 8.1.0; GraphPad Software, La Jolla, CA, USA). Values are shown as rounded means with standard deviation where applicable. Except for the combined viability/ALP activity assay (n = 6) and the CAM assay (Table 2), each measurement was performed in n = 5 biological replicates.

## Results

### BG characterization and ion release kinetics

**Figure 1:**
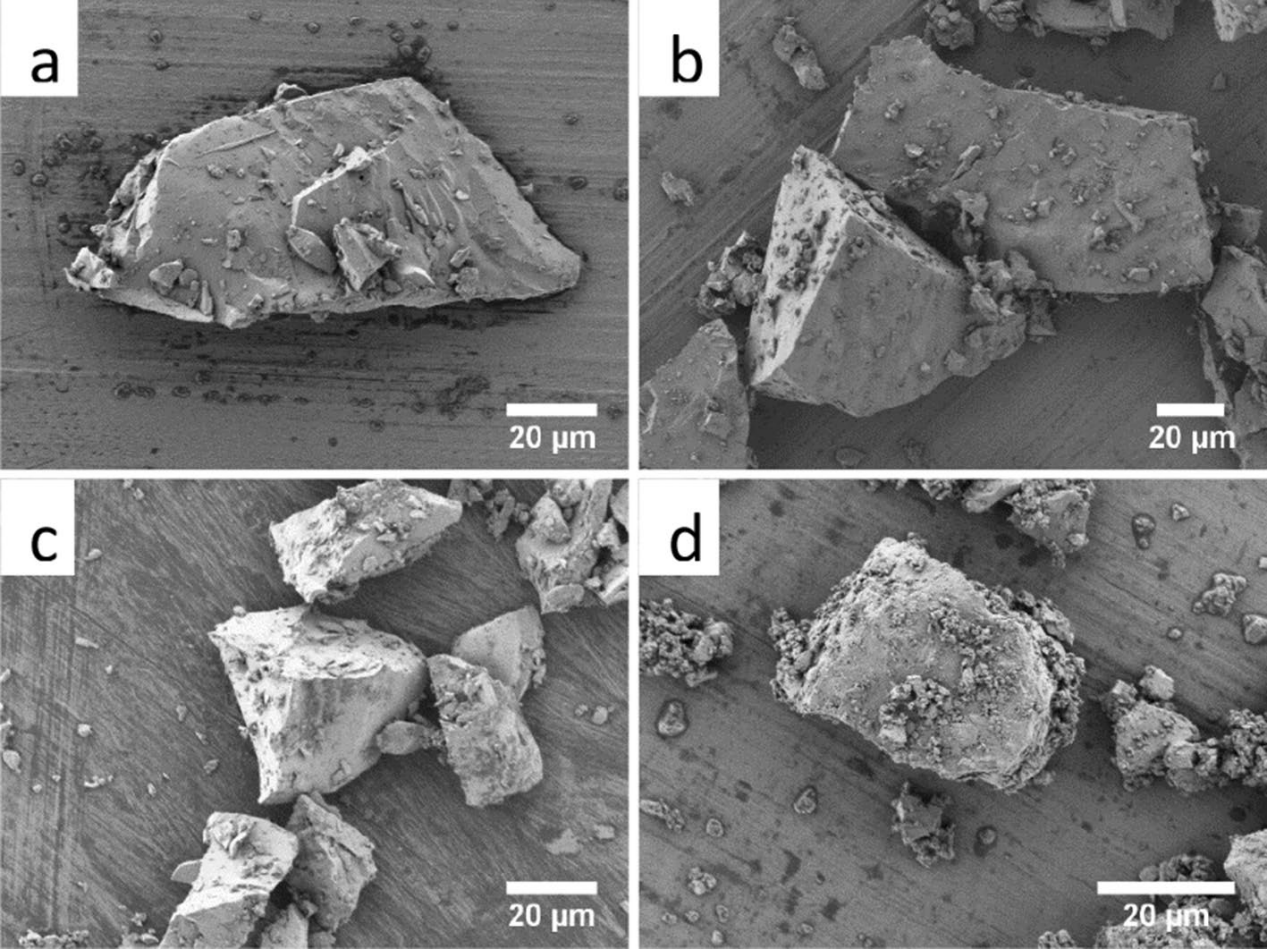
Scanning electron microscopy (SEM) showing the morphology of the sintered BG particles investigated in this study, namely (**a**) ICIE16-BG, (**b**) 3B-BG, (**c**) 4B-BG and (**d**) 8B-BG.

The sintered BG particle’s morphology was examined using SEM, showing their polyhedral morphology (Fig. 1). The estimated particle sizes were 71 ± 28, 80 ± 23, 67 ± 17 and 53 ± 25 µm for ICIE16-BG, 3B-BG, 4B-BG and 8B-BG, respectively.

**Figure 2:**
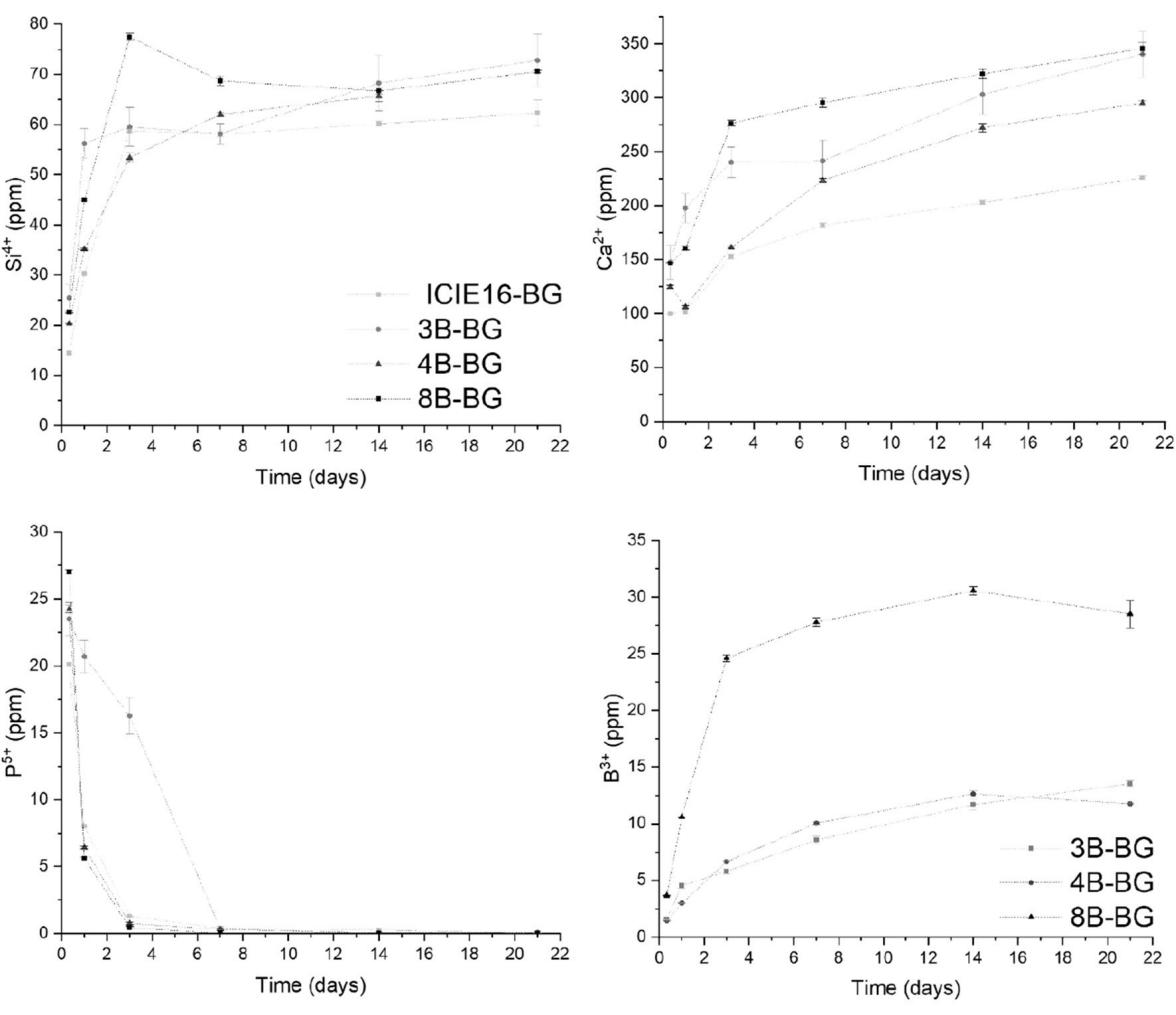
Silicon (Si^4+^), calcium (Ca^2+^), phosphorus (P^5+^) and boron (B^3+^) ion release from the investigated undoped and B-doped BGs incubated in simulated body fluid (SBF), as measured by ICP-OES.

The concentrations of leaching ions from the BG particles in SBF are shown in Fig. 2. The increasing concentration of silicon ions (Si^4+^) in the medium suggests the dissolution of the BGs occurring from the first hours of incubation until reaching a steady concentration after 3 days. 8B-BGs showed the highest dissolution rate. The decrease of the phosphorus (P^5+^) concentration might be an indication of the precipitation of calcium-phosphate phases on the glass particles, with this process being slower for the 3B-BG particles compared to the other samples. Regarding the release of B ions (B^3+^), a fast release occurred during the first 3 days of incubation in all BG groups, followed by an increasing, but slower release until 7 days and a steady behavior after 14 days. There was no significant difference between the amount of B^3+^ ions detected from the 3B-BG and 4B-BG samples, while 8B-BGs released a clearly higher amount of B^3+^ ions.

### IDPs of ICIE16-BG and B-doped BGs decreased cell viability

**Figure 3:**
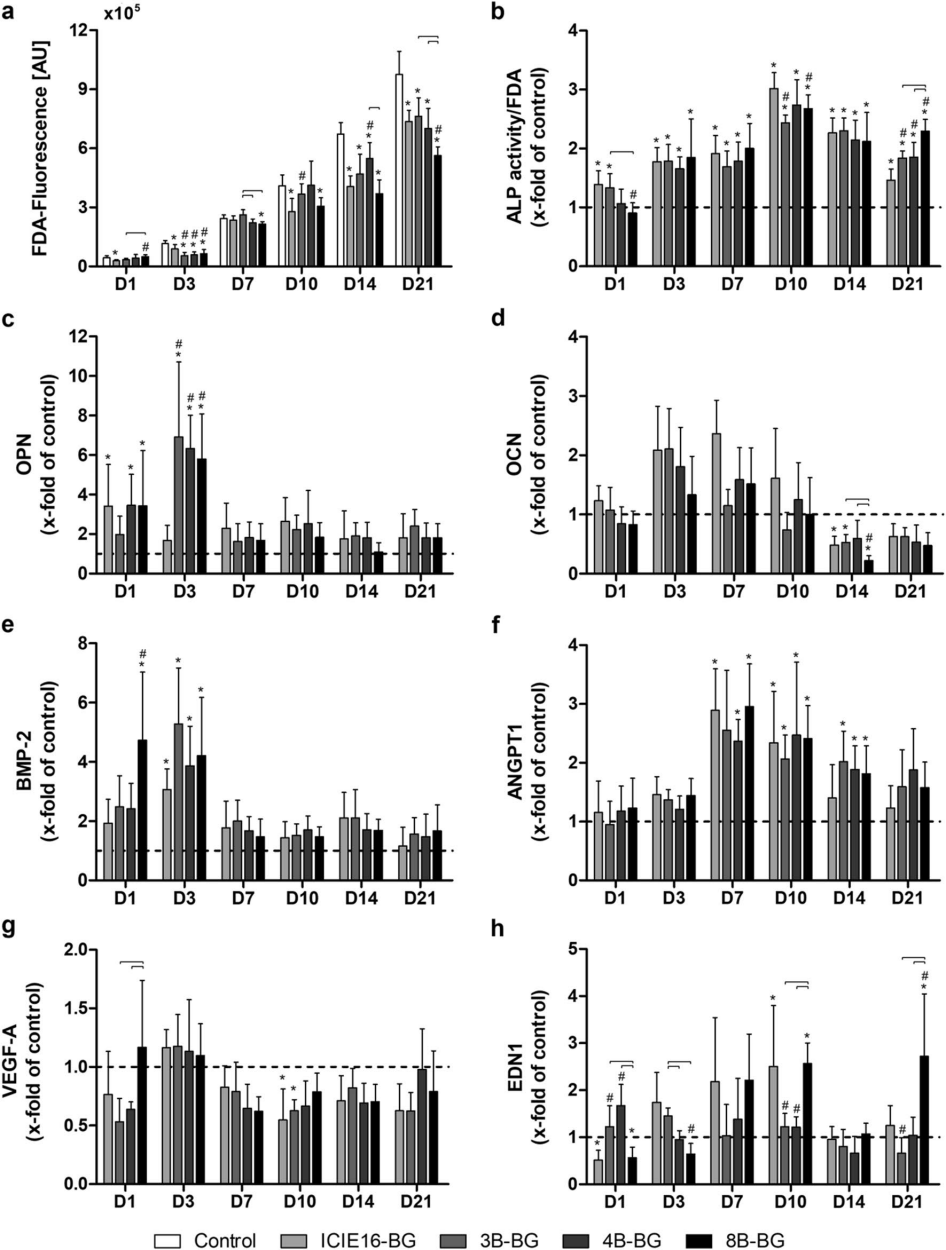
Viability of BMSCs (**a**), ALP activity of BMSCs (normalized to FDA) (**b**), expression of osteogenic marker genes, namely OPN (**c**), OCN (**d**) and BMP-2 (**e**) and expression of angiogenic marker genes, namely ANGPT1 (**f**), VEGF-A (**g**) and EDN1 (**h**). Except for viability measurements (**a**), values are normalized to the control group indicated by the dashed line (**b–h**). [*] marks significant differences compared to the control group, [#] marks significant differences compared to ICIE16-BG. Significant differences between the B-doped BGs are highlighted with brackets.

Compared to the control group, IDPs of all BGs negatively affected BMSC viability (Fig. 3a). IDPs of ICIE16-BG decreased cell viability to a significant extent on all days except for D7, but especially from D10 on. The influence of the IDPs of the B-doped BGs was less negative: While IDPs of 3B- and 4B-BG initially diminished BMSC viability, it was increased above the ICIE16-BG group’s level or at least comparable from D7 on. IDPs of 4B-BG showed the most pronounced effect, since cell viability was comparable to the control group on D10 and significantly above the ICIE16-BG group on D14. Although IDPs of 8B-BG enhanced cell viability compared to the other BG groups on D1, viability levels were comparable to the ICIE16-BG group in the following days and significantly declined on D21.

### Influence of the BGs’ IDPs on cell morphology and viability

**Figure 4:**
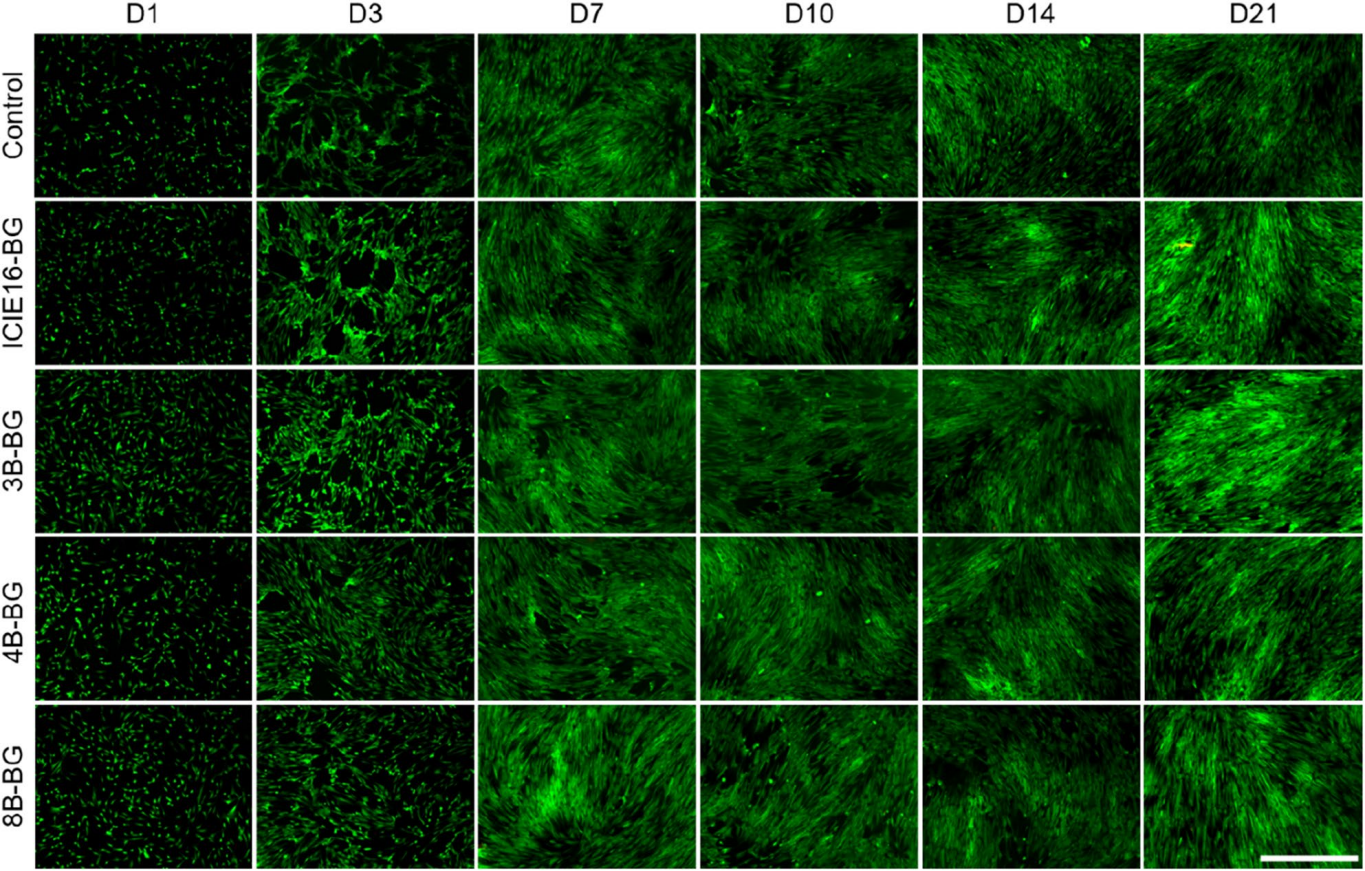
Representative live/dead-assay for the indirect culture setting and the control group after an incubation time of 1 (D1), 3 (D3), 7 (D7), 10 (D10), 14 (D14) and 21 (D21) days. Viable cells are shown in green, compromised cells in red. Scale bar (bottom right corner) refers to 1000 μm and applies to all images. Magnification: 40-fold.

On D1, the density of green-stained cells in the B-doped BG groups seemed to be approximately the same, but higher compared to the control and ICIE16-BG groups (Fig. 4). Cell density in the control and ICIE16-BG groups seemed to increase on D3, thus matching the quantitative FDA measurements. Since cells already reached confluency in all groups after 7 days, no remarkable differences or increases in cell density could be observed thereafter. Only very few red-stained compromised cells were detectable in general, as dead detached cells are likely to be removed during the washing steps of the staining procedure.

### IDPs of undoped and B-doped BGs enhanced cellular osteogenic differentiation and osteogenic marker gene expression

IDPs of all BG groups had a positive impact on ALP activity (Fig. 3b). While IDPs of ICIE16-BG and 3B-BG significantly increased ALP activity above the level of the control on D1, the other B-doped BGs did not show clear effects, as ALP activity remained on the control’s level. From D3 on, ALP activity was significantly enhanced in all BG groups when compared to the control group. No clear benefit of B-doping regarding ALP activity was observable from D3 to D14, whilst all B-doped BG variants significantly outperformed the undoped ICIE16-BG on D21, with 8B-BGs also outperforming all other B-doped groups. OPN expression was elevated above the level of the control group in all BG groups at all measured time points (Fig. 3c). While IDPs of ICIE16-BG, 4B-BG and 8B-BG significantly upregulated OPN expression compared to the control group on D1, all B-doped BGs showed significantly higher expression levels compared to the control and ICIE16-BG groups on D3. No remarkable differences between the groups were observable from D7 on. OCN expression was upregulated by IDPs of ICIE16-BG from D1 to D10, while IDPs of the B-doped BGs showed no clear influences, as OCN expression was upregulated on D3 and D7 but declined thereafter (Fig. 3d). On D14, expression levels dropped significantly under the control group’s level in all BG groups except for 4B-BG: Whilst IDPs of 3B-BG and especially 4B-BG seemed to have a less negative effect than ICIE16-BG, IDPs of 8B-BG led to a significantly lower OCN expression than in any other group. OCN expression remained under the control’s level in all BG groups on D21. All BG groups showed higher BMP-2 gene expression levels compared to the control group at all measured time points (Fig. 3e). While IDPs of 8B-BG had the most pronounced effect on BMP-2 expression on D1, leading to a significantly higher expression compared to the control and ICIE16-BG groups, all BG groups showed a positive influence on D3, resulting in significant upregulations. In the following days, no significant differences among the groups were observed.

### B-doping had a moderately positive influence on angiogenic marker gene expression

The BGs’ IDPs showed little influence on ANGPT1 gene expression during the first three days (Fig. 3f). A distinct upregulation of ANGPT1 expression was observed in all BG groups from D7 on. IDPs of ICIE16-BG significantly enhanced expression levels on D7 and D10, but decreased in the following days, being the lowest amongst the BG groups from D14 on. IDPs of B-doped BGs mostly upregulated ANGPT1 expression to a significant extent throughout D7 and D10 as well, and the expression remained significantly increased until D14. VEGF-A expression was barely affected by the IDPs of the different BGs (Fig. 3g). IDPs of 8B-BG seemed to positively influence VEGF-A expression compared to the other BG groups on D1. Expression levels were comparable between all groups on D3, followed by a slight downregulation in all BG groups in the following days. No clear differences between the BG groups were observed. The impact of the BGs’ IDPs on EDN1 expression was very mixed (Fig. 3h): Whilst IDPs of ICIE16-BG initially had a negative effect on EDN1 expression, an upregulation was observed from D3 to D10, followed by expression levels comparable to the control group. IDPs of 3B- and 4B-BG seemed to have a positive influence from D1 to D3, but expression levels remained on the level of the control group thereafter. The influence of IDPs of 8B-BG was mostly comparable to ICIE16-BG until D14, but a remarkable upregulation of EDN1 expression above all other groups was observed on D21.

### CAM assay: ‘survival-rate’ and ‘take-rate’ of cell transplants

The ‘survival-rate’ of CAMs seeded with the BMSC-IDPs-mixture was 91.8%, whereas the rate of CAMs with visible cell transplants on D7, termed as ‘take-rate’ was 69.4% (Table 3). Embryos that died before EDD 16 were excluded from the study, as well as CAMs without a visible cell transplant (Table 3). Two samples were identified as extreme values (values deviating ≥ 3 interquartile ranges from the median) via boxplot method and therefore excluded. The number of biological replicates included in the quantitative analysis of the angiogenic response was at least n = 4 per group (Table 3).

**Table 3:** Number of biological replicates in the CAM assay at different time points: directly after transplantation (D0), after explantation (D7), with visible cell transplants (microscopic evaluation) and after exclusion of extreme values. Data in brackets describe the individual dropouts for each group and time point. Data in brackets written in *italics* describe the overall relative rate (in %) of included biological replicates, equivalent to the ‘survival-rate’ at D7 and to the ‘take-rate’ of transplants after microscopic evaluation.

	number of biological replicates			
group	at D0 (EDD 9)	at D7 (EDD 16, explantation)	with visible cell transplant (microscopic evaluation)	after exclusion of extreme values
Control	10	10 (0)	9 (1)	7 (2)
ICIE16-BG	9	7 (2)	4 (3)	4 (0)
3B-BG	10	8 (2)	6 (2)	6 (0)
4B-BG	10	10 (0)	8 (2)	8 (0)
8B-BG	10	10 (0)	7 (3)	7 (0)
Total	49 *(100%)*	45 *(91.8%)*	34 *(69.4%)*	32 *(65.3%)*

### IDPs of B-doped BGs significantly enhanced angiogenesis in ovo with increasing B-concentration

**Figure 5:**
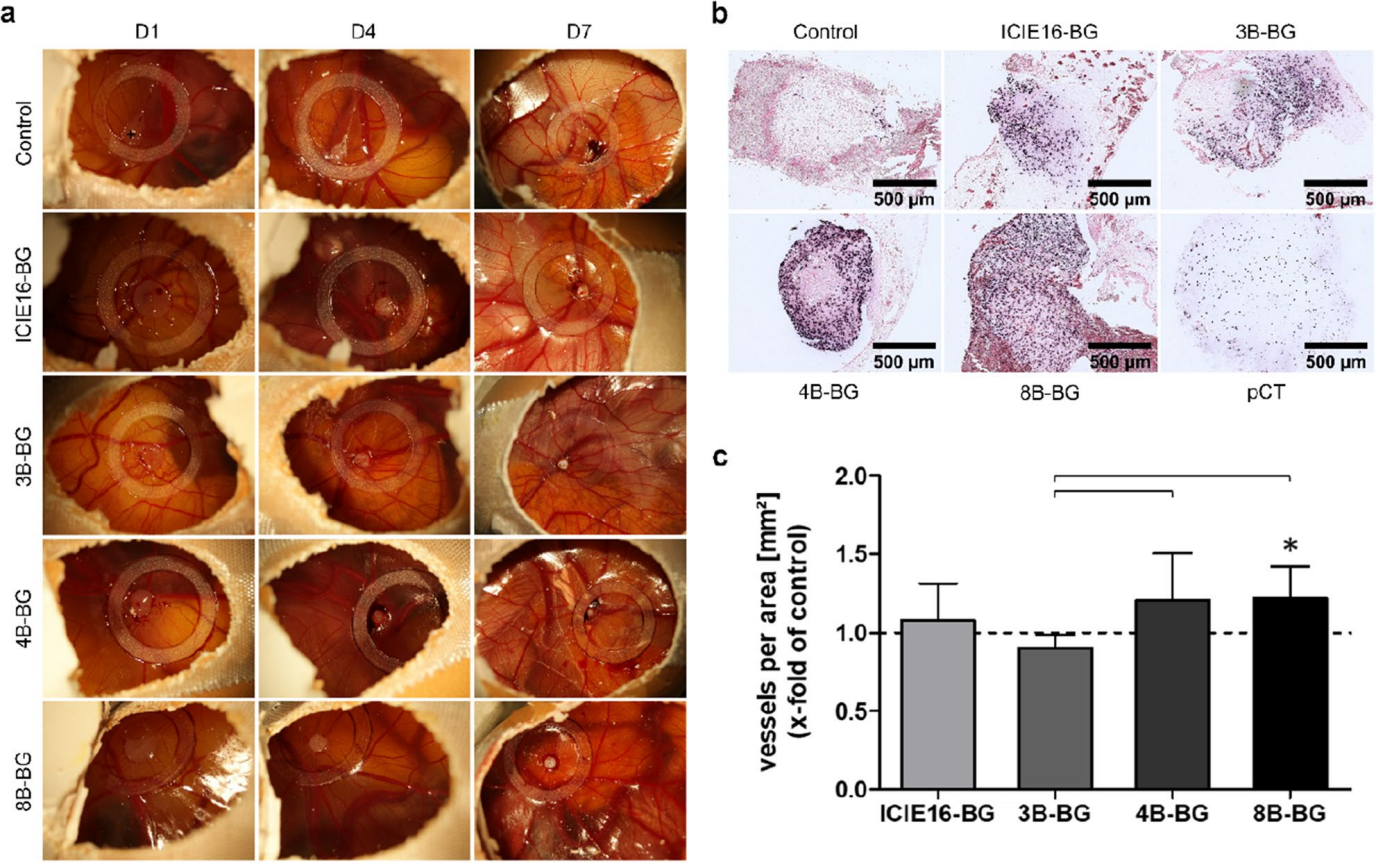
(**a**) Representative macroscopic images of the CAM assay for the control group and the BG groups after an incubation time of 1 (D1), 4 (D4), and 7 (D7) days post transplantation. Cell transplant (transparent/white) exemplarily marked with [ +] in the first picture. The inner diameter of the silicone rings refers to 9 mm and was used as size reference. (**b**) Representative microscopic images of the in situ hybridization of CAM resectates after a seven-day incubation period for the control group and the BG groups. Human BMSCs were present in all groups, as indicated by dark staining of nuclei (in a range from violet to black) following hALU-labeling. To prove a correct function of the in situ hybridization, a positive control (pCT; BMSC pellet), was assessed as well. (**c**) Vessels per area 7 days (D7) post transplantation. Values are normalized to the control group indicated by the dashed line. [*] marks significant differences compared to the control group. Significant differences between the B-doped BGs are highlighted with brackets.

While the number of visible vessels increased in all groups from D1 to D7 as seen in qualitative macroscopic assessment of the CAM assay, the size of the cell transplants decreased (Fig. 5a). However, BMSCs were detectable via in situ hybridization in all examined groups after explantation, thus confirming viability of cell transplants throughout the in ovo incubation period (Fig. 5b). No remarkable differences regarding vascularization were observed between the groups in macroscopic pictures.

IDPs of ICIE16-BG slightly increased the number of vessels per area compared to the control group (Fig. 5c). IDPs of 3B-BG had a negative influence compared to the control group and all other BG groups since the number of vessels per area was significantly lower than both other B-doped BG groups. IDPs of 4B-BG and 8B-BG increased the number of vessels per area compared to the control and ICIE16-BG groups, with the 8B-BG group being also significantly above the control group’s level.

## Discussion

Compared to the 45S5-BG, ICIE16-BG shows favorable processing properties: it exhibits a low crystallization tendency when sintered due to a greater thermal processing window, which is suitable to produce amorphous (non-crystalline) BG scaffolds by powder sintering exhibiting homogeneous dissolution process^46,47^. Therefore, incorporating ions into the structure of ICIE16-BG represents an attractive approach to benefit from the specific therapeutic effects provided by the ions^7,48^. In a previously published study by our group, biocompatibility and osteogenic properties of 45S5-BG and ICIE16-BG were directly compared in vitro^5^. ICIE16-BG showed the expected biocompatibility, in fact viability was slightly higher in the ICIE16-BG group compared to the 45S5-BG group^5^. Furthermore, a positive influence of ICIE16-BG on the cellular osteogenic differentiation was observed^5^. By doping BGs with therapeutically active ions, specific tailoring of the BGs’ properties can be achieved: in earlier studies, B-doped BGs showed good osteogenic and pro-angiogenic features^22,25,27,29^, making B an attractive dopant ion.

In this study, three different B-doped ICIE16-based BGs, namely 3B-BG, 4B-BG and 8B-BG were evaluated regarding their biocompatibility, as well as their influence on osteogenic differentiation and angiogenesis. An indirect cultivation setting was used to assess the impact of B-doped BGs on cells that are not directly adjacent to the material, but only exposed to the BGs’ IDPs^36,37^. The study was divided into an in vitro and an in ovo part. In the in vitro part, the main subject was the analysis of the impact of the BGs’ IDPs on BMSC viability, cellular osteogenic differentiation and expression of osteogenic marker genes. Furthermore, the impact of the BGs’ IDPs on angiogenesis was analyzed, as vascularization is not only essential for physiological bone growth and remodeling, but also for bone defect healing^49,50^: When critical-sized bone defects are treated with mesenchymal stem cells, the size of the regenerated bone is limited due to lacking vessels in the grafts, as stated by He et al.^51^. For bone defect healing, it is therefore crucial that angiogenesis and osteogenesis go hand in hand, also termed as ‘angiogenic-osteogenic coupling’^18^. Hence, this study focused on the analysis of potential pro-angiogenic properties of the B-doped BGs by evaluating the expression of angiogenic marker genes in vitro and, furthermore, investigation of the IDPs’ influence on angiogenesis in a CAM assay in ovo.

BMSC viability was negatively affected by IDPs of all examined BGs—however, BGs with a low to moderate release of B^3+^ ions, like 3B- and 4B-BGs, had a slightly positive influence on cell viability compared to IDPs of ICIE16-BG. Comparable results were described by Durand et al.^27^: An indirect cultivation setting was used in their study to investigate the influence of IDPs of 45S5.2B-BG, a 45S5-BG doped with 2 wt% B on HUVECs^27^. After incubating HUVECs with IDPs for 48 h, IDPs of 45S5.2B-BG outperformed IDPs of 45S5-BG regarding cell proliferation^27^. In a study conducted by Wu and co-workers, B-doped mesoporous BGs increased proliferation of human osteoblasts in a positive concentration-dependent manner, compared to undoped BGs^24^. When BMSCs were cultured with IDPs of 8B-BG, releasing high amounts of B^3+^ ions, viability levels were comparable to the ICIE16-BG group at first but declined significantly on D21, which might be explained via a continuously high release of B ions from the 8B-BG. Negative influences of high B^3+^ concentrations on cell viability were also described by Brown et al.: In their study, examining the influence of B-doped 45S5-BGs on MC3T3-E1 cells, a mouse pre-osteoblastic cell line, cell proliferation was found to decrease with increasing B content in BGs^21^. When B content in BGs was very high, massive decreases in cell viability were reported by Balasubramanian and co-workers in their study on the influence of the IDPs of different B-doped BGs on mouse bone-marrow derived ST-2 cells^29^. At the same time, moderate and low B content had no negative or even positive effects on cell viability^29^. Hence, there seems to be a specific therapeutic window in which B-doped BGs exhibit a good biocompatibility, whereas cytotoxic effects occur when B concentrations are too high, explaining the significant drop in cell viability observed in the 8B-BG group on D21 in the present study. Brown et al. described a borate ion concentration of 2.5 mM (which refers to 27.03 mg/l ionized B) as critical, as they observed a reduction in cell proliferation greater than 50% upon culture with MC3T3-E1 cells^21^. In our study, the B release from the 8B-BG peaked at D14, reaching a concentration of around 30 mg/l, exceeding the critical level of toxicity as defined by Brown et al.^21^**.** However, when defining a B-caused reduction in viability of more than 50% as cytotoxic, the B release from 8B-BG can be considered as non-cytotoxic since BMSC viability was still higher than 50% of the control group. This observation indicates a potentially higher resistance of BMSCs towards B-mediated toxicity as extensive differences in cytotoxicity of BGs depending on cell types have been observed before^52^. However, it has to be considered that the ion release analyses of the B-doped BGs were conducted in SBF whilst DMEM was used for the cell culture assays. The known impact of different media on the BGs’ ion release cannot be estimated and therefore constrains the interpretation of our observations^48^. Discussing the negative influence of high B concentrations on BMSC viability becomes particularly important in regard to the known potential negative impact of B. Cytotoxicity represents one of the most important effects, which is quite relevant as it might limit other potentially positive influences of B-doping^9^. Moreover, it is reported that high B concentrations can lead to (systemic) abnormalities regarding development and reproduction in rats as well as in other species^9,12^. However, there is not much information on toxic B levels in humans, which seem to be extremely high^12^.

To assess the influence of the BGs’ IDPs on osteogenic differentiation, ALP activity, a well-known marker enzyme for early osteogenic differentiation^53,54^, as well as the expression of the osteogenic marker genes OPN, OCN and BMP-2 was analyzed^55–57^. While no clear benefit of B-doping regarding ALP activity was observable initially, IDPs of B-doped BGs outperformed the undoped ICIE16-BG on D21. Culturing cells with B-containing IDPs had a mostly positive impact on osteogenic marker gene expression, however, compared to IDPs of undoped BGs, only slight upregulations were observed. Most studies reported positive effects of B-doped BGs on osteogenic differentiation as well: Houaoui et al. studied the influence of scaffolds consisting of Polylactic acid (PLA) and either B-doped or undoped BGs on myoblastic C2C12 cells^58^. They found an upregulation of osteogenic differentiation in the B-BG group, measured through OPN immunostaining^58^. Furthermore, Ojansivu and co-workers studied the influence of S53P4-based B-doped BGs on human adipose stem cells (hASCs) in an indirect culture setting in vitro^59^. Interestingly, they reported a decrease in ALP activity with increasing B concentrations, while osteogenic marker gene expression was enhanced similarly^59^. Doping of 45S5-BG with 2 wt% B enhanced bone formation in a rat tibia model in vivo, as reported by Gorustovich et al.^25^. While most studies postulated a remarkable positive impact of B-doped BGs on osteogenic differentiation, only slightly positive pro-osteogenic effects were observed in the present study. In addition to the use of different cell types and culture settings, other factors might explain the reported differences regarding the pro-osteogenic features of B-doped BGs and their IDPs, such as the specific BG that was doped with B, assessment methods or assessed time points.

The influence of the BGs’ IDPs on angiogenesis was evaluated via expression of relevant angiogenic marker genes in vitro, as it is known that BMSCs promote angiogenesis via paracrine signaling^60,61^. Assessed genes include VEGF-A, a potent inducer of neovascularization^62^, ANGPT1, well-known for its function in mediating neovessel maturation^63^ and EDN1, a potent vasoconstrictor but, nevertheless, also pro-angiogenic factor mostly renowned for its role in tumor angiogenesis ^64^. A distinct upregulation of ANGPT1 expression was observed in all BG groups from D7 on. Whereas B-doped BGs seemed to prolongate this upregulation, VEGF-A expression was barely affected by the BGs in general. The impact of the BGs’ IDPs on EDN1 expression was very mixed, but IDPs of 8B-BG had a positive influence from D10 on. In summary, the pro-angiogenic effects exhibited by the IDPs of B-doped BGs in vitro can be designated as limited. The influence of B-doped BGs on VEGF secretion of ST-2 cells was investigated in different studies by Balasubramanian et al., working in a direct culture setting^29^ and Chen et al., working with BGs’ IDPs in an indirect culture setting^28^. Interestingly, VEGF release decreased upon culture with BGs (or their respective IDPs) containing high B contents compared to undoped BGs in both studies^28,29^, whilst similarly lower B concentrations increased VEGF secretion in a direct culture setting^28^ or had neither positive nor negative effects in an indirect culture setting^29^, matching the results of the present study. Thus, there seems to be a specific therapeutic window for B-doped BGs to enhance VEGF expression and secretion, which appears to be independent of the silicate BG matrix involved. However, a clearly pro-angiogenic impact of B-doped 45S5-BGs was reported in an in vitro study by Durand et al., as migration and tubulogenesis of HUVECs were enhanced compared to undoped BGs^27^, contrasting the limited pro-angiogenic effects of B found in the in vitro part of the present study. Furthermore, borosilicate glass 0106-B1 scaffolds seeded with BMSCs significantly outperformed 45S5-BG scaffolds regarding VEGF-A expression in an in vivo study with mice^22^. While the comparison of studies using different cell types and different cultivation settings is quite difficult, as mentioned earlier^20,52^, it is also important to recognize that angiogenesis and vascularization become increasingly important when researched models of higher complexity are considered, as in 3D cell culture models or in vivo experiments^16–18^.

Hence, the influence of the B-doped BGs’ IDPs on angiogenesis was further evaluated in ovo in a CAM assay that has been established for the use with BMSCs especially for the purposes of this study. Since CAM assays are mostly used to analyze drug effects on angiogenesis or in cancer research, no evidence regarding the seeding of BMSCs directly onto the CAM was found. Thus, characteristic key data, such as ‘survival-rate’ and ‘take-rate’ were used to prove whether the CAM assay is suitable for usage with BMSCs. While Kunz and co-workers reported a ‘survival-rate’ of approximately 80% and a ‘take-rate’ of 94% when working with invasive-growing osteosarcoma cells^41^, compared to ‘survival- ‘ and ‘take-rates’ of 91.8% and 69.4% in the present study, Mangir et al. reported *ex ovo* embryo ‘survival-rates’ of 68% for intermediate and 83% for experienced investigators in an acellular setting^65^. Therefore, the CAM assay proves to be suitable for the use as angiogenesis assay with BMSCs, especially since human cells were detectable on D7 via in situ hybridization of repetitive species-specific genomic sequences. Also, when compared to the popular HUVEC tube formation assay, the CAM assay warrants comparable results when applied to one and the same setting as shown by Hsieh and coworkers^66^. IDPs of 4B-BG and 8B-BG clearly increased the number of vessels per area compared to the control and IDPs of undoped ICIE16-BG, while IDPs of 3B-BG showed a negative influence. Evidence regarding the influence of B-doped BGs or their IDPs on angiogenesis in a CAM assay is very rare, as only one relevant study conducted by Durand and co-workers was found^17^. They observed similar results, when investigating the influence of IDPs of 45S5.2B-BG, a B-doped BG on angiogenesis in an acellular setting on the embryonic quail CAM^17^: IDPs of 45S5.2B-BG enhanced the number of blood vessel branching points compared to IDPs of undoped BGs^17^. Moreover, they reported that upon addition of borate (without BGs/IDPs), angiogenesis was increased in a positive concentration-dependent manner, going well in line with the findings of the present study^17^. Thus, higher B concentrations seem to have a positive impact on angiogenesis in ovo.

In summary, IDPs of B-doped BGs showed only slightly pro-angiogenic effects in vitro, but (with increasing B-concentrations) a positive influence on angiogenesis in ovo. In the used in vitro 2D cell culture setting, oxygen and nutrients supply is provided by diffusion, whereas in experimental settings with increasing complexity, such as in vivo models, the role of vascularization and angiogenesis becomes increasingly important^67^. This might possibly explain the observed limited effects of the B-doped IDPs on angiogenesis in the in vitro part of the present study, whilst at the same time angiogenesis was significantly promoted in ovo. The impact of the IDPs of B-doped BGs on osteogenic differentiation of BMSCs was also mainly positive, yet not very pronounced. Comparable results were observed in a study previously published by our group: In vitro, the impact of 45S5-BG and the B-doped 0106-B1-BG on the osteogenic differentiation of BMSCs was comparable, while 0106-B1-BG scaffolds significantly outperformed 45S5-BG scaffolds in terms of osteogenesis and angiogenesis in a mouse model in vivo^22^. Hence, it seems to be of importance to establish new, innovative models for biomaterial research, that can be categorized between the classic in vitro and in vivo models. This might allow to overcome the limitations of 2D in vitro cell culture settings and similarly avoid excessive use of sophisticated and resource-intensive in vivo animal testing. Moreover, these models should focus on enabling a better insight into ‘angiogenic-osteogenic coupling’. Besides the CAM assay, 3D cell culture models^68^ or combined cell culture settings, for instance with BMSCs and HUVECs^69^, as well as vasculature-on-a-chip models or bioreactors^70^ are possible options. Furthermore, application of the CAM assay for analyzing the influences of BGs or their respective IDPs on osteogenesis represents an attractive option for future studies also to identify promising candidate BGs to be introduced in actual in vivo bone defect models after detailed investigation, both in vitro and in ovo.

## Conclusions

Whilst the influence of IDPs of B-doped BGs on BMSC viability was dose-dependent, with lower B concentrations showing slightly positive and higher B concentrations showing slightly negative influences, IDPs of B-doped BGs had a moderately positive impact on osteogenesis and angiogenesis in vitro. In contrast to that, B-doping showed clearly positive influences on angiogenesis in ovo, especially in higher concentrations. The differences between the results of the in vitro and in ovo part of this study might be explained via the different importance of vascularization in the used settings. Thus, establishing new, innovative models that can be categorized between the classic in vitro and in vivo models and enable better insight into ‘angiogenic-osteogenic coupling’ is highly relevant, for instance via application of a CAM assay for analysis of the influences of BGs or their respective IDPs on osteogenesis.

## Data availability

The datasets generated and analyzed during the current study are available from the corresponding author on reasonable request.
